# Evaluating the efficacy of Rose Bengal-PVA combinations within PCL/PLA implants for sustained cancer treatment

**DOI:** 10.1007/s13346-024-01711-w

**Published:** 2024-09-23

**Authors:** Sara Demartis, Camila J. Picco, Eneko Larrañeta, Anna Korelidou, Rayhanul Islam, Jonathan A. Coulter, Paolo Giunchedi, Ryan F. Donnelly, Giovanna Rassu, Elisabetta Gavini

**Affiliations:** 1 Department of Chemical, Physical, Mathematical and Natural Sciences, University of Sassari, Sassari, 07100 Italy; 2 School of Pharmacy, Queen’s University Belfast, Belfast, BT9 7BL UK; 3 Department of Medicine, Surgery and Pharmacy, University of Sassari, Sassari, 07100 Italy

**Keywords:** Rose Bengal, Polyvinyl alcohol, Cancer, Implant, 3D printing, Sustained drug release

## Abstract

The current investigation aims to address the limitations of conventional cancer therapy by developing an advanced, long-term drug delivery system using biocompatible Rose Bengal (RB)-loaded polyvinyl alcohol (PVA) matrices incorporated into 3D printed polycaprolactone (PCL) and polylactic acid (PLA) implants. The anticancer drug RB’s high solubility and low lipophilicity require frequent and painful administration to the tumour site, limiting its clinical application. In this study, RB was encapsulated in a PVA (RB@PVA) matrix to overcome these challenges and achieve a localised and sustained drug release system within a biodegradable implant designed to be implanted near the tumour site. The RB@PVA matrix demonstrated an RB loading efficiency of 77.34 ± 1.53%, with complete RB release within 30 min. However, when integrated into implants, the system provided a sustained RB release of 75.84 ± 8.75% over 90 days. Cytotoxicity assays on PC-3 prostate cancer cells indicated an IC50 value of 1.19 µM for RB@PVA compared to 2.49 µM for free RB, effectively inhibiting cancer cell proliferation. This innovative drug delivery system, which incorporates a polymer matrix within an implantable device, represents a significant advancement in the sustained release of hydrosoluble drugs. It holds promise for reducing the frequency of drug administration, thereby improving patient compliance and translating experimental research into practical therapeutic applications.

**Supplementary Information:**

The online version contains supplementary material available at 10.1007/s13346-024-01711-w.

## Graphical Abstract

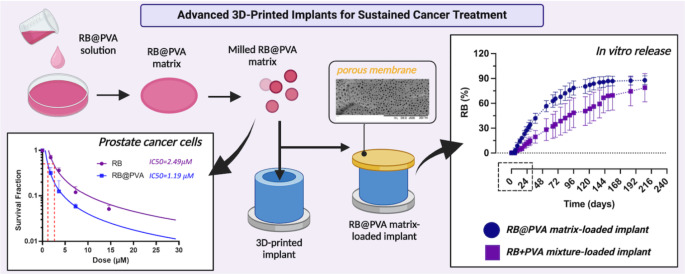

## Introduction

Projections for 2024 predict cancer cases will exceed 2 million for the first time, underscoring the urgent need for more effective treatments. This translates to nearly 5,500 new diagnoses and over 1,600 deaths daily, highlighting the critical public health challenge. The rise in cancer cases can be attributed to population ageing and increased diagnosis of significant cancers, such as breast, prostate, endometrial, pancreatic, kidney, and melanoma [1]. This trend emphasises the need for enhanced prevention, early detection, and treatment strategies to effectively manage the rising cancer burden.

Despite impressive advances, there remains a persistent need for novel cancer treatments that overcome current obstacles, including nonspecific drug action and side effects. While surgical resection is ideal for localised tumours, it is limited by its unsuitability for specific conditions and the risk of leaving behind microscopic disease [2]. This may necessitate additional treatments such as radiotherapy, which, despite its potential, can cause significant long-term side effects and does not work equally for all patients [3]. Furthermore, adjuvant chemotherapy, often used when surgery and radiotherapy are insufficient or infeasible, especially for inoperable tumours, suffers from severe side effects due to the nonspecific action of chemotherapeutic agents. The lack of specificity for many anticancer drugs causes challenges concerning achieving effective drug concentrations at the tumour site, further complicated by narrow therapeutic indices and poor pharmacokinetic and pharmacodynamic characteristics of many anticancer drugs [4–7]. Therefore, developing advanced drug delivery systems is crucial for more effective and less toxic cancer treatments. Localised and controlled drug delivery systems would ensure that drugs are deposited directly at the tumour site, reducing unwanted systemic exposure and improving patient outcomes.

Recent advancements in local drug delivery have included using various materials and technologies to optimise drug release profiles and biocompatibility. For instance, 3D-printed implants have emerged as a versatile platform for personalised medicine, allowing precise control of drug release to the tumour site. This thereby markedly diminishes the side effects on surrounding healthy tissues and optimises the therapeutic efficacy through controlled drug release mechanisms, potentially preventing the requirement for frequent therapeutic interventions [8–12]. Among the implantable devices reported in the literature, implants fabricated using 3D printing techniques are particularly attractive since a wide range of parameters can be adjusted to obtain a device with macro and micro characteristics matching specific requirements, for instance, the dimension and shape of tumour cavity, or the desired drug release rate [13–16].

Regarding the composition of drug delivery systems, biomaterials are the leading choice for enhancing treatment efficacy with minimised side effects [8, 17–20]. Polyvinyl alcohol (PVA) has been widely studied in this field due to its biocompatibility and customisable properties based on the facile manipulation of its degree of hydrolysis and polymerisation [21–23]. PVA demonstrated excellent biocompatibility owing to its non-toxic, non-carcinogenic, and non-immunogenic characteristics, allowing PVA-based materials to interact favourably with biological tissues and fluids [24]. Regarding biodegradability, enzymatic processes facilitate the decomposition of PVA into water and carbon dioxide in aqueous environments. PVA’s degradation rate can be personalised as it depends on its molecular weight and degree of hydrolysis [25–27].

Rose Bengal (RB), a xanthenic molecule, shows remarkable anticancer activity against multiple cell lines, including colon, breast, oral, ovarian, pancreas, gastric, head, and melanoma cancers. RB may act through two mechanisms: its photo-sonosensitizing properties and intrinsic cytotoxicity. Indeed, the presence of the xanthenic ring in the RB structure is responsible for its activation when triggered by light or ultrasound stimuli, which consequently permits its application in photodynamic and sonodynamic therapy [28, 29]. At the same time, the parallel intrinsic cytotoxicity of RB has been evidenced; RB is likely to preferentially accumulate in malignant cell lysosomes, causing cell lysis finally. This subsequently triggers a second response based on an immune reaction. However, RB does not lack shortcomings [30, 31]. The reduced half-life (30 min) and the induction of severe systemic toxicity, related to its biopharmaceutical profile and photosensitising properties, limit the application of RB [32–34]. Currently, the intralesional injection of an aqueous solution of RB (PV-10^®^) is the only administration route in cancer treatment [35]. However, repeated administrations are required to reach the therapeutic window of RB, exposing patients to painful procedures and needing constant medical assistance.

This research introduces the development and characterisation of an advanced delivery system designed for sustaining RB release in local cancer treatment. To address the challenge posed by RB’s high water solubility, a medicinal matrix loading RB (RB@PVA) was fabricated using PVA and incorporated into a biomaterial-based 3D printed implant to modulate the drug release rate [36]. PVA was selected for its known safety and versatility. The implant device, previously developed and characterised by the same authorship, demonstrated exceptional biocompatibility in tests with human umbilical vein endothelial cells [37]. With its favourable cytotoxic profile, biodegradability, and controlled drug release capabilities, this implant aligns with the requirements for an effective RB delivery system that ensures spatial and temporal release control, enhancing patient compliance. In conclusion, this research proposes an innovative RB administration route to reduce administration frequency significantly compared with its counterpart, PV-10^®^. RB-loaded implantable devices are envisioned to be surgically implanted near the tumour site without requiring surgical removal at the end of the therapy. Furthermore, their application could be considered an adjuvant therapeutic after surgical resection of the primary tumour or a standalone treatment.

Finally, building on our previous work with PCL/PLA implants, this study presents a novel system for the extended release of highly water-soluble drugs. Unlike our earlier research, which focused on less soluble compounds, this work incorporates a polymer matrix within the implant to modulate release kinetics effectively. This approach directly addresses the challenges of controlling the release of hydrosoluble drugs, marking a significant advancement in the development of implantable drug delivery systems. Furthermore, this innovative strategy holds promise for enhancing the translational aspects of drug delivery, potentially bridging the gap between experimental research and practical therapeutic applications.

## Materials and methods

### Materials

#### Chemicals

Rose Bengal sodium salt (RB) of ≥ 95% purity and polyvinyl alcohol (PVA) 87–90% hydrolysed (MW = 30–70 kDa) were purchased from Sigma-Aldrich (Dorset, UK). Poly(caprolactone) (PCL) polymers, CAPA™ 6506 (MW = 50 kDa) and CAPA™ 2054 (MW = 550 Da) were obtained from Ingevity (South Carolina, USA). Granulated Poly(lactic acid) (PLA) polymers, Ingeo™ 3D850 (MW = 190 kDa), Ingeo™ 4043D (MW = 200 kDa), and an additional PLA variant (MW = 160 kDa), were obtained from NatureWorks (Minnesota, MN, USA). Dichloromethane (DCM) was sourced from Merck (Darmstadt, Germany).

#### Cell line and reagents

PC-3 prostate cancer cells were used as a cancer cell line model. They were obtained from the American Type Culture Collection (ATCC) and grown in Roswell Park Memorial Institute (RPMI) medium (Thermo Fisher Scientific) supplemented with 10% foetal bovine serum (FBS, Gibco). All routine culturing was performed at 37 °C and 5% CO2/95% air. Short tandem repeat profiling and mycoplasma testing (Lonza) were routinely conducted for this cell line.

### RB long-term stability

RB’s long-term stability was evaluated in a buffer medium (KH_2_PO_4_ / NaOH, pH 7.4). A 5% w/v RB buffered solution was prepared and incubated in the dark at 37 °C for 5 months. The storage conditions were selected considering the proven photodegradation of RB, with the temperature replicating that of the human body [32]. At each predetermined time point, the solution was analysed for absorbance (Abs) by UV-Vis spectrophotometry (Shimadzu UV-1800, Kyoto, Japan), exciting the maximum absorption wavelength (λ_max_ = 549 nm) of RB in the medium. The RB stability was assessed based on Abs variation and the RB amount (mg) over time. A calibration curve in water was employed for calculating RB (mg) amount (calibration standards in the 1–20 mg/L range; y = 0.10640119x + 0.00531086; R^2^ = 0.999).

### Fabrication of the implant

**Fig. 1:**
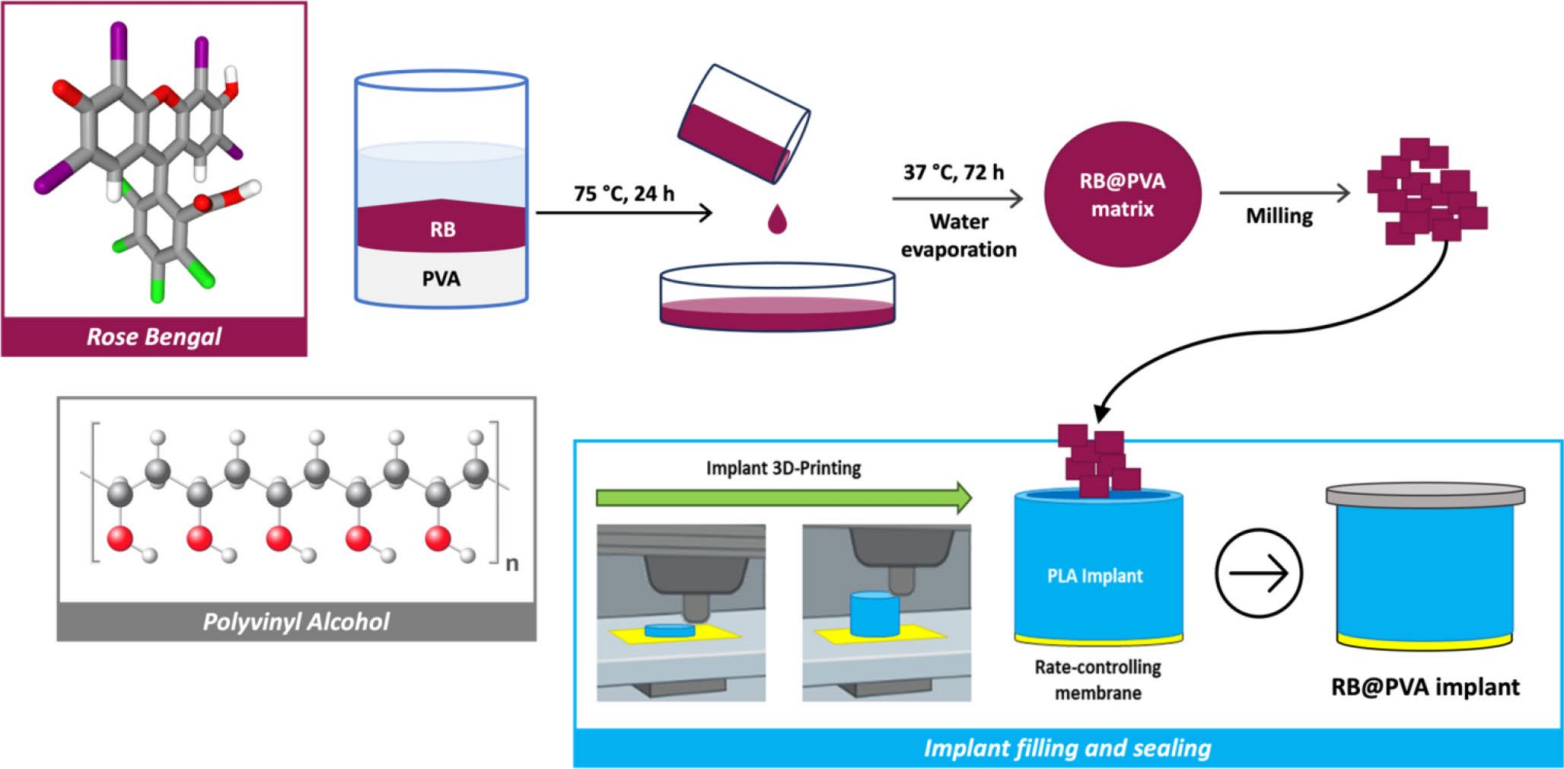
Illustration of the RB-PVA (RB@PVA) matrix preparation using the casting-solvent evaporation technique and its inclusion into the 3D-printed implant. RB and PVA powders were dissolved in deionised water, and the viscous solution was cast into a glass petri dish. The dried matrix was removed and milled. The milled RB@PVA matrix was included in the 3D-printed implant. For implant fabrication details, refer to Korelidou et al., 2022 [37]. Symbols: RB- Rose Bengal, PVA- Polyvinyl Alcohol

Implants consisted of hollow cylinders with a permeable membrane at one end to control drug release and an impermeable cover (sealing) at the other end (Fig. 1). The implants were fabricated using the 3D printing technique of fused deposition modelling (FDM) (Ultimaker 3, 3D printer), as reported in our previous investigation [37].

Briefly, a 50:50 mixture of PCL (MW = 550 Da) and PLA (MW = 190 kDa) was used to prepare the polymeric porous membrane. These membranes were fabricated via the solvent casting method, using DCM as the solvent at a 3% w/v polymer concentration. The membranes were then positioned on the build plate of the FDM 3D-printer to be incorporated into the implant structure. PLA filaments (MW = 200 kDa) were employed to print the cylindrical implants directly onto these 50PCL:50PLA membranes, resulting in one side of the implant being covered with a porous, semi-permeable membrane. After printing, the implants were manually filled with RB formulations. To create a non-porous, non-permeable barrier, the opposite end of the cylindrical implant was sealed with a manually applied molten layer of PCL (MW = 50 kDa).

### Preparation of the RB-loaded PVA (RB@PVA) matrix

RB@PVA matrix was prepared using a casting-solvent evaporation technique. Initially, an RB/PVA viscous solution was obtained by dissolving RB (150 mg) and PVA (3 g) powders in 15 mL of deionised water and placed in an oven at 75 °C for 24 h to ensure complete solubilisation [38, 39]. The following day, the RB/PVA viscous solution was cast in a glass petri dish (7.5 cm in diameter), with excess water evaporated at 37 °C for 72 h. The resulting matrix was removed from the petri dish with a spatula, then frozen for 10 min at -80 °C and subjected to a blade milling process (MF 10 basic, IKA^®^ WERKE) for 2 min at 4000 rpm to obtain milled RB@PVA matrix. The resulting particles larger than 1 mm were collected using a sieve with a 1 mm mesh and then incorporated into the implant, with each implant filled with 10 mg of milled RB@PVA matrix (Fig. 1).

### Characterisation of RB@PVA matrix

#### RB quantitative determination

The milled RB@PVA matrix was evaluated for RB quantitative determination regarding the theoretical drug content (Theor. DC%), experimental drug content (Exp. DC%) and loading efficiency (LE%). Theor. DC% was calculated as follows:

1 $$\:Theor.\:DC\%=\frac{RB\:\left(mg\right)\:weighed}{RB\:\left(mg\right)\:weighed+\rm{PVA}\:\left(mg\right)\:weighed}\times100\:$$

Exp. DC% was calculated as follows:

2 $$\:Exp.\:DC\%=\frac{RB\:\left(mg\right)\:in\:RB@\rm{PVA}\:matrix}{Weighed\:of\:RB@\rm{PVA}\:matrix\:\left(mg\right)}\times100$$

For quantification in the RB@PVA matrix, 10 mg of the formulation was dispersed in methanol (50 mL) as an extraction solvent and magnetically stirred until RB extraction was complete (70 °C, 300 rpm, 3 h). Samples were centrifuged at 4000 rpm for 10 min (Eppendorf Centrifuge 5702R, Eppendorf, Milan, Italy) to precipitate PVA, which is insoluble in methanol. The supernatant was decanted, and RB content was measured by UV-Vis spectrophotometry (Shimadzu UV-1800, Kyoto, Japan) and calculated by referencing a calibration curve in methanol (calibration standards in the range of 0.7–11.8 µg/mL; *y* = 0.1214x-0.0456; *R*^2^ = 0.9997). Lastly, the LE% was expressed as $\:\frac{Exp.\:\:DC\%}{Theor.\:\:DC\%}\times100$.

#### Morphology and particle size determination

The milled RB@PVA matrix was visually examined by optical microscopy using a Leica EZ4W stereomicroscope (Leica Microsystems, Milton Keynes, UK) and a scanning electron microscope (SEM) (TM3030, Hitachi, Krefeld, Germany). For SEM experiments, the milled RB@PVA matrix was deposited onto adhesive carbon tape and observed under vacuum with a voltage of 15 kV without sample pre-treatment. The lengths of 16 representative RB@PVA particles were measured directly from the microscopy images to determine the particle size distribution. These measurements were used to calculate the mean particle size, standard deviation, and size range in accordance with European Pharmacopoeia (Ph. Eur.) guidelines.

#### Mechanical testing

The mechanical properties of RB@PVA and pure PVA matrices were tested using TA. XT plus texture analyser (Stable Micro Systems, Surrey, UK) at a constant extension speed of 10.2 mm/min and 0.049 N force. Strips measuring 4 cm in length, 1 cm in width, and 0.2 mm in thickness were prepared by cutting the samples with a calliper. The strips were clamped with an inter-clamp distance of 10 mm, with force-displacement curves recorded. The elastic modulus was calculated as the slope of the first linear section of the stress/strain curve. The ultimate tensile strength (UTS) and elongation at failure were calculated from the stress/strain curves of the tested strips.

#### Differential scanning calorimetry (DSC) and attenuated total reflectance Fourier Transform-Infrared spectroscopic analysis (ATR-FTIR)

DSC and ATR-FTIR analyses were performed for the samples of RB@PVA matrix, RB/PVA physical mixture, RB powder, PVA powder, and pure PVA matrix. DSC was conducted using a Q100 differential scanning calorimeter (TA Instruments, Bellingham, WA). Scans were run from 25 °C to 350 °C with a heating rate of 10 °C/min under a nitrogen flow rate of 50 mL/min. A Spectrum Two FT-IR Spectrometer (Perkin Elmer, Waltham, MA) was used along with MIRacle™ Single Reflection attenuated total reflectance (ATR) (Pike Technologies, Fitchburg, MA) with a MIRacle™ Confined Space Clamp to analyse potential interactions between the drug and polymers, for Spectra were recorded from 4000 to 600 cm^− 1^ with a resolution of 4 cm^− 1^. All the spectra presented in this work were obtained from an average of 32 scans.

#### In vitro dissolution study of milled RB@PVA matrix

For the evaluation of the dissolution profile of milled RB@PVA matrix, 10 mg were dispersed in 10 mL of buffer medium (pH 7.4) and incubated at 37 °C in an orbital incubator (SKI 4 Shaker Incubator, Argo Lab, Carpi, Italy) at 40 rpm. At each predetermined time point (5, 10, 15, 20, 30, 60 min), the entire medium was collected and filtered under vacuum using a Büchner funnel with a cellulose membrane filter with pore size in the magnitude of micrometres. The isolated residual RB@PVA matrix was frozen at -80 °C, then freeze-dried (LIO-5P 4 K, Milan, Italy) for 7 h. Afterwards, the residual RB@PVA matrix was weighed. The percentage of matrix weight loss and the corresponding solubilised matrix were calculated at each time point (details on calculations are reported in the supplementary material). To ensure the accuracy and reliability of the weight loss data, all measurements were conducted under tightly controlled conditions to minimise potential sources of error. This included maintaining a stable environment with consistent temperature and using standardised sample handling and weighing procedures to ensure reproducibility. The accuracy of the measurements was further validated by repeating the weight loss measurements multiple times (*n* = 4).

To evaluate the RB in vitro release profile, 10 mg of RB@PVA was dispersed in 10 mL of buffer medium and incubated at 37 °C at 40 rpm. At each predetermined time point (5, 10, 15, 20, 25, 30, 35 min, and 1 h), 0.5 mL of medium was collected for RB quantification and immediately replaced with fresh medium. The study was conducted under sink conditions. RB was measured using a UV-Vis spectrophotometer and calculated using the calibration curve prepared in water. The cumulative amount of RB released over time was calculated. For comparison, the in vitro release profile was also conducted for the physical mixture (10 mg) of PVA and RB powders (RB/PVA PM) containing the same RB amount of RB@PVA matrix.

### In vitro RB release study from RB@PVA matrix-loaded implants

The in vitro RB release profile from milled RB@PVA matrix-loaded implants was evaluated in buffer solution (pH 7.4). Each implant was filled with 10 mg of RB@PVA matrix and placed in a 10 mL medium. Samples were incubated at 37 °C at 40 rpm. At regular intervals, the entire medium was collected for RB quantification and immediately replaced with an equal amount of fresh medium to maintain consistent release conditions and prevent the buildup of RB aggregates in the solution. RB concentration in the collected samples was measured by UV-Vis spectrophotometry, referencing the calibration curve in water. The cumulative amount of RB released over time was calculated and compared across different implant formulations (*n* = 4). For comparison, the in vitro release study was also conducted for implants filled with RB/PVA PM (10 mg) containing the same RB amount of RB@PVA matrix and for implants filled with RB powder alone (RB = 2 mg). The study was conducted under sink conditions. At the end of the experiment, the implants were opened, and the permeable membrane was analysed by SEM.

### Cytotoxicity study on prostate cell lines

Clonogenic assays were performed according to our previously published reports [40]. In brief, 1 × 10^5^ cells were plated in a 6-well plate and allowed to adhere. After a 24 h incubation, cells were treated with different doses of solubilised RB or RB@PVA matrix, ranging from 1.88 µM to 30 µM in a complete culture medium for 24 h. Untreated control and vehicle-only controls (PVA at a dose of 30 µM) were also included. These concentration ranges were selected based on the results of the in vitro release studies from RB@PVA matrix-loaded implants. Excess RB, RB@PVA matrix, or PVA was removed, and cells were washed twice with PBS and incubated in complete media before replating. Cells were trypsinised, diluted 1:1 in fresh medium, and vortexed to ensure a homogenous solution before being counted and reseeded at low densities for clonogenic assay. After 14 days, colonies were fixed in 0.4% crystal violet and 70% methanol and then counted using a 50-cell exclusion criterion. Colonies with more than 50 cells were counted to determine plating efficiency (PE), defined as the number of colonies formed divided by the number of seeded cells. Surviving fractions (SF) were then calculated relative to untreated control. The difference between the untreated and vehicle control was adjusted for the RB@PVA matrix-treated groups.

### Statistical analysis

All numerical data herein reported are expressed as mean ± standard deviation (SD) of at least *n* ≥ 3 experimental measures, calculated using GraphPad Prism^®^ 10.0.0 (153) (GraphPad^®^ Software, San Diego, USA). The same software was used to analyse the data set regarding statistical significance. The statistical method employed for singular data sets is indicated in Sect. 3. A p-value < 0.05 was considered statistically significant. Significance level is graphically indicated: *p-value < 0.05, **p-value < 0.01, ***p-value < 0.001, ****p-value < 0.0001.

## Results and discussion

This study focuses on advancing drug delivery techniques to enhance the efficacy and specificity of cancer treatments. A novel strategy involving an RB-loaded PVA matrix incorporated into a 3D-printed implantable device explicitly designed for localised therapy is proposed to achieve this. The device performance and potential to address this challenge were evaluated through comprehensive in vitro experiments, the outcomes of which are reported below.

### RB long-term stability

RB was evaluated for stability over 5 months at 37 °C in the dark, simulating physiological conditions. This study aimed to assess whether RB maintained chemical integrity over this extended period, which is crucial for justifying its use in drug delivery systems for sustained release. In this experiment, the RB concentration was 5% w/v, corresponding to 50 mg/mL, selected based on the amount of RB loaded into the RB@PVA matrix (about 5% w/w).

**Fig. 2:**
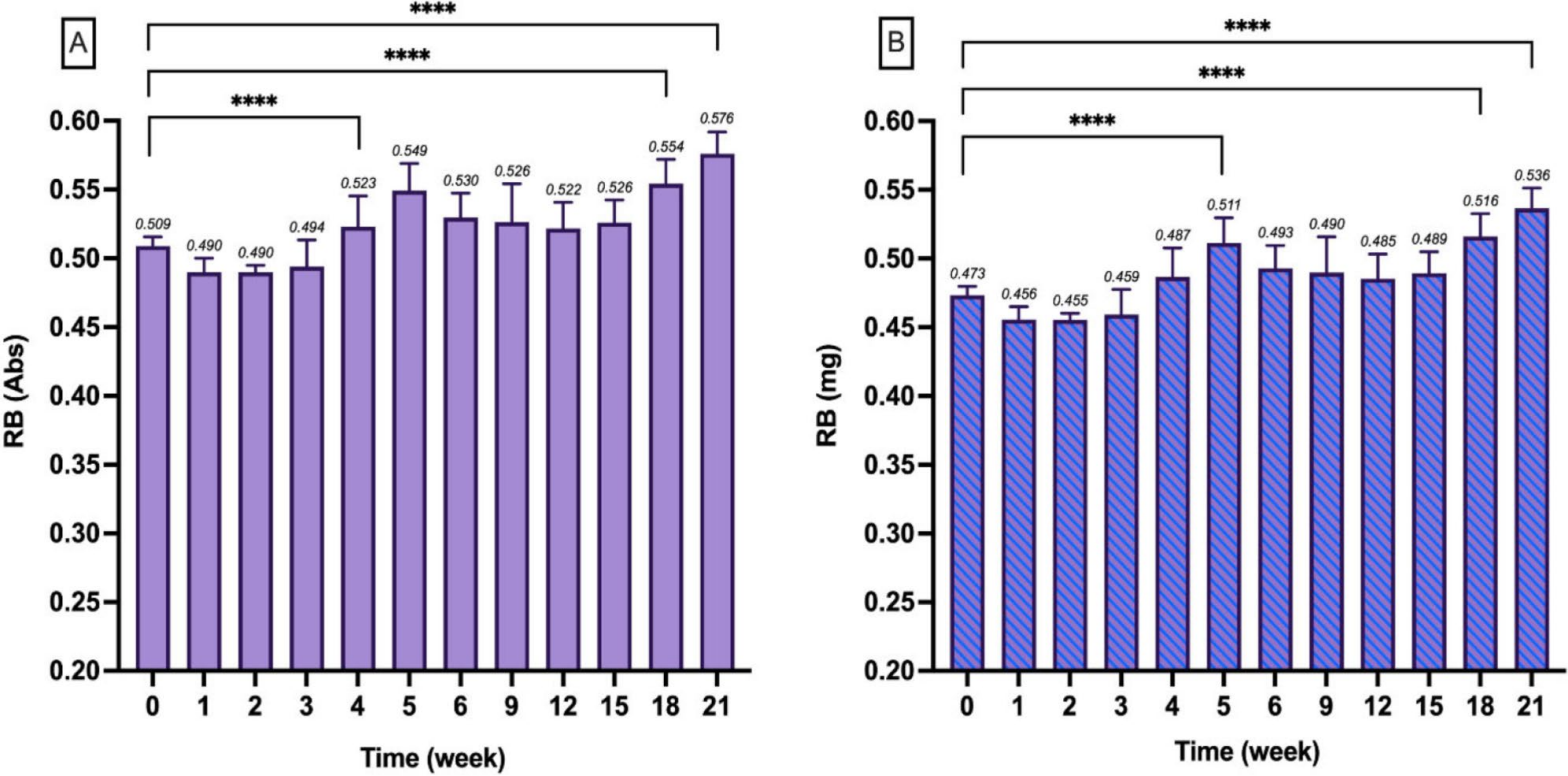
Long-term stability of Rose Bengal (RB) in buffer solution (pH 7.4) assessed by absorbance (Abs) at 549 nm (**A**) and converted in mg (**B**) over time. Results are expressed as mean ± SD of three samples, each analysed in triplicate (*n* = 9). Statistical significance was determined using Dunnett multiple comparison tests with a single pooled variance, comparing each point with the baseline (week 0). Significance is indicated as ****p < 0.0001

The results, shown in Fig. 2, indicated that the Abs values of the RB solution remained relatively stable over the 5 months, suggesting no significant degradation. Nevertheless, there was a slight increase in the Abs values, potentially indicating RB molecule aggregation, with significance recorded at 5, 18, and 21 weeks (p-value < 0.0001 vs. starting time). This aggregation might have led to the clustering of RB molecules, resulting in higher local concentrations detected by the spectrophotometer. The study by Alvarez-Lopez et al. reports that RB aggregation, particularly into H-type aggregates, leads to a decrease in Abs peak due to the formation of dimers or higher-order aggregates. Conversely, the current investigation observed an increase in Abs. This increase may be due to altered light absorption characteristics of these aggregates, a phenomenon also noted in other reports [41]. Studies by Das et al. and Mohammed et al. support this observation, indicating that forming H-type aggregates at higher concentrations can result in unique optical properties and increased Abs [42, 43]. Importantly, through the stability study, the λ_max_ of RB remained consistent at 549 nm, suggesting that, despite the aggregation, the RB retained its monomeric form during the measurements. Finally, the percentage variation of the reported RB (mg) values during the stability study was 3.03±6.16% compared to the initial value, indicating that the overall chemical integrity of RB was maintained.

Regarding the photochemical-related applications of RB (e.g., photodynamic therapy (PDT)), this bis-anionic dye’s tendency to aggregate limits RB’s use in solution because the dimeric form does not possess photosensitising properties [44–46]. However, it is essential to note that this aggregation issue, particularly relevant to PDT applications, does not impact the intrinsic cytotoxicity of RB, the mechanism exploited in this investigation. Moreover, while this accumulation is observed in vitro at the highlighted concentration, in vivo conditions involve metabolism and elimination processes likely to prevent such aggregation and accumulation.

### Preparation and characterisation of RB@PVA matrix

RB@PVA matrix was prepared using a bio-friendly solvent-casting technique with ultrapure water as the exclusive solvent. Due to the paramount importance of assuring the safest possible drug delivery system for cancer applications, water-soluble excipients were selected to prevent the use of potentially harmful solvents during the formulation process [47, 48]. Based on this and the considerations outlined in the introduction, PVA was chosen as a bulk polymer for the matrix. Moreover, the molecular weight of the PVA selected for this study falls within the range of polymers that can be excreted via renal filtration [49].

**Fig. 3:**
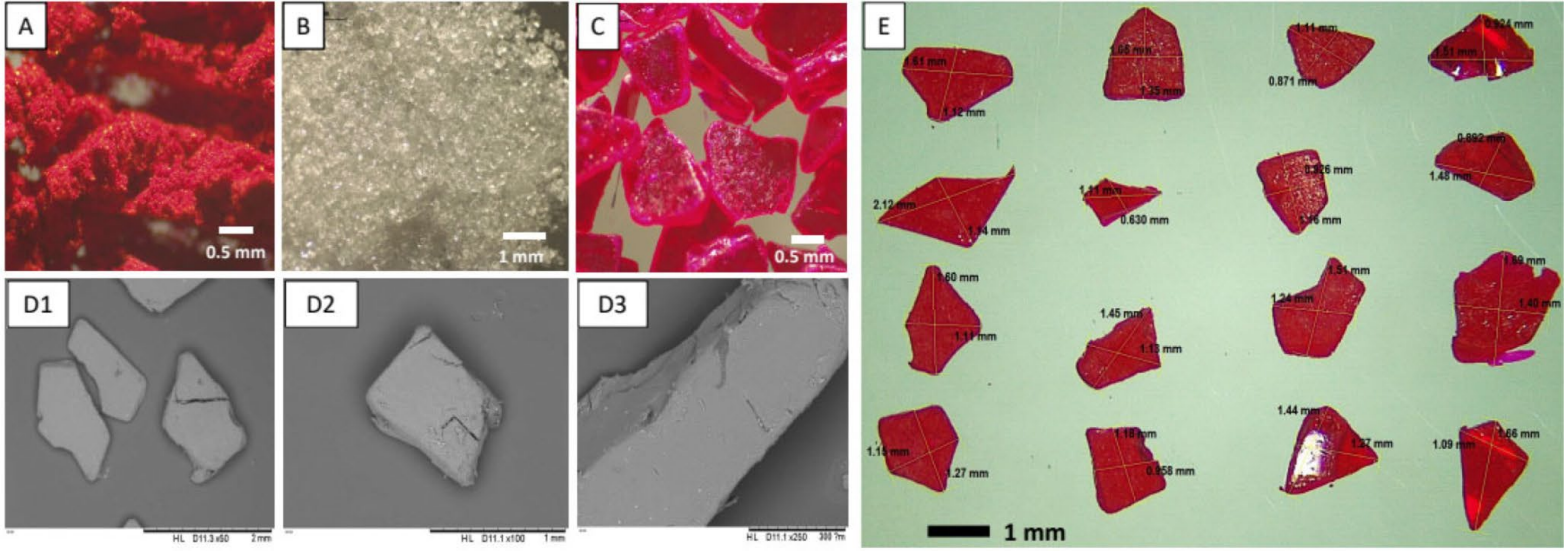
Morphology and size distribution of the RB-loaded PVA (RB@PVA) matrix after milling. **(A)** RB powder under optical microscopy (scale bar 0.5 mm, magnification 25x). **(B)** PVA powder under optical microscopy (scale bar 1 mm, magnification 16x). **(C)** Milled RB@PVA matrix under optical microscopy (scale bar 0.5 mm, magnification 25x). (**D1-3**) Scanning electron microscopy (SEM) images of milled RB@PVA matrix (D1: scale bar: 2 mm, magnification: 50x; D2: scale bar: 1 mm, magnification: 100x; D3: scale bar: 300 μm, magnification: 250x). (**E**) Size measurement of 16 RB@PVA particles

RB@PVA matrix was easy to prepare using the named technique. As the final step of formulation, the RB@PVA matrix was milled, and particles ranging from 0.5 to 2.0 mm in size (both height and length) were obtained (Fig. 3). The particle size distribution analysis revealed that the particles had a mean length of approximately 1.06 ± 0.29 mm and a mean height of 1.23 ± 0.25 mm, indicating some degree of variability. The size of RB@PVA particles was intentionally maintained in the millimetre range to prevent a fast drug release that could happen in the case of nano- or micrometre-sized particles [50]. Moreover, the specific range of about 0.5–2.0 mm facilitates the loading of particles in the cavity of the implant, which possesses a diameter of approximately 7 mm [37].

The RB content in the milled product was determined to be 36.8 mg per gram of the final product, providing a clear and practical measure of the drug loading efficiency. RB loading efficiency (LE%) was 77.34 ± 1.53%, consequent to a Theor. DC% of 4.76% and an Exp. DC% of 3.68 ± 0.07%. The loss of approximately 1% RB between Theor. DC% and Exp. DC% was attributed to the challenges of casting the entire RB/PVA viscous solution from the beaker to the petri dish (Fig. 1).

**Fig. 4:**
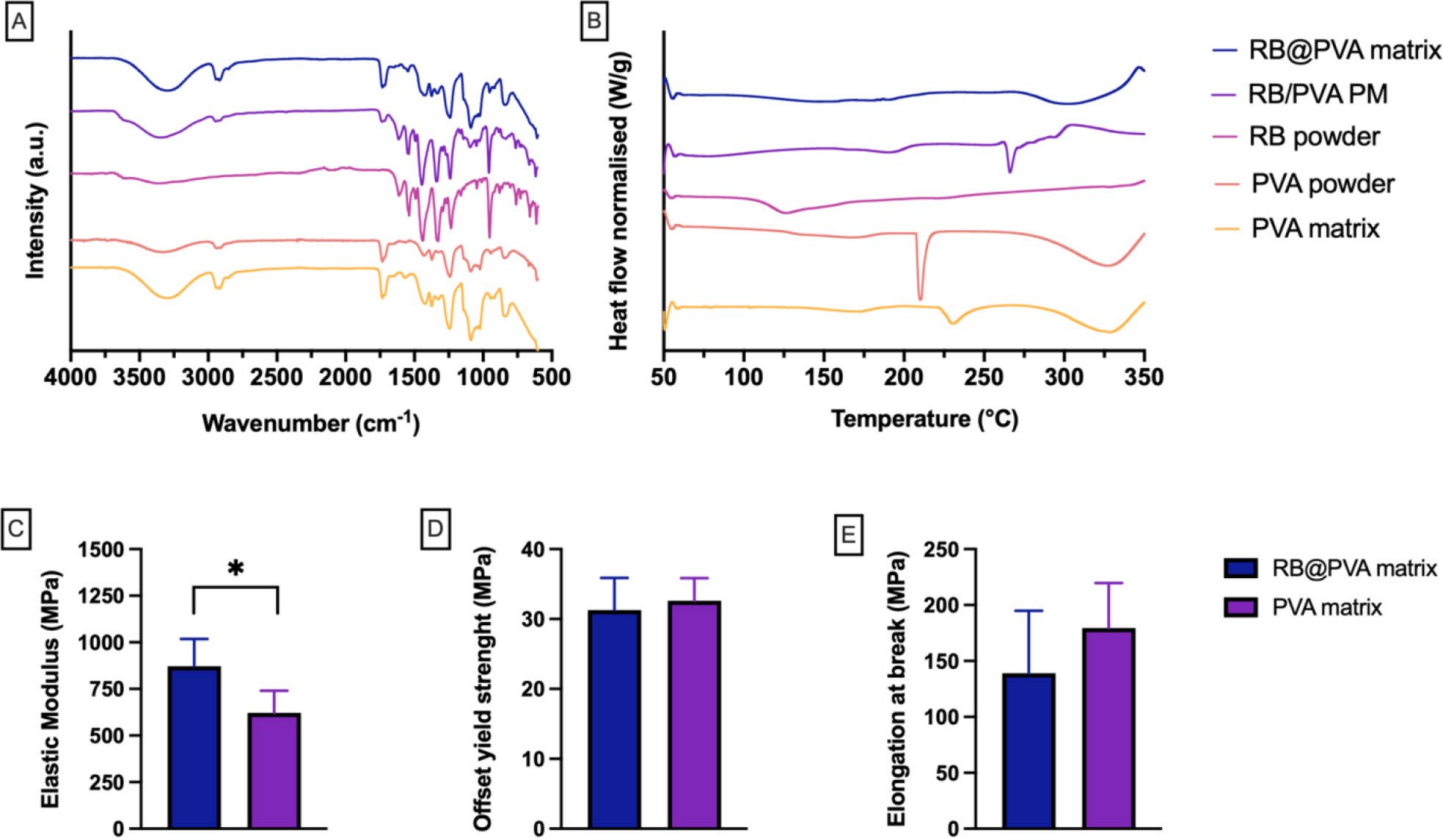
Physicochemical and mechanical characterisation of RB-loaded PVA (RB@PVA) matrix. **(A)** FTIR spectra of RB powder, PVA powder, RB/PVA physical mixture, and RB@PVA matrix showing characteristic peaks (transmittance over wavenumber range (4000–600 cm^− 1^)). **(B)** DSC thermograms of RB powder, PVA powder, RB/PVA physical mixture, and RB@PVA matrix (heat flow over temperature (°C)). **(C)** Elastic modulus. **(D)** Offset yield strength. **(E)** Ultimate tensile strength of pure PVA matrix and RB@PVA matrix. **C-E**: Results are expressed as mean ± SD of four samples (*n* = 4). Statistical analysis was performed using an unpaired t-test (two-tailed). Significance is indicated as *p < 0.05, **p < 0.01

The physicochemistry of the RB@PVA matrix was analysed using FTIR (Fig. 4A) and DSC (Fig. 4B).

The DSC results of pure RB showed a broad peak starting around 100 °C, which can be attributed to the loss of moisture from the sample. On the other hand, pure PVA exhibited a clear peak above 200 °C, which can be attributed to the melting point of PVA [51]. This peak can be found in the pure PVA matrix prepared using the same procedure used to prepare the RB@PVA matrix. However, the peak obtained is less intense and broader, suggesting a reduction in crystallinity. Conversely, the analysis of the RB@PVA matrix did not show a precise melting point for PVA, indicative of interactions between RB and PVA.

Interestingly, the RB/PVA physical mixture exhibited a small broad peak attributed to PVA melting at around 200 °C. The RB/PVA physical mixture revealed a different thermal behaviour to that of the RB@PVA matrix, confirming the interactions suggested earlier between RB and PVA. FTIR analysis of pure RB and PVA powders presented characteristic peaks for these substances. Pure PVA showed a broad peak at around 3300 cm^− 1^, attributed to the OH group, and a characteristic C = O peak due to acetyl groups at around 1700 cm^− 1^ [51]. On the other hand, RB produced a characteristic C = O band at around 1600 cm^− 1,^ and a band around 950 cm^− 1^ for OH stretch. The physical mixture of PVA and RB produced a combination band characteristic for the pure compounds, while the RB@PVA matrix exhibited mainly peaks corresponding to PVA.

Furthermore, some small RB peaks can be observed in the RB@PVA matrix spectra at around 950 cm^− 1^ and around 1550 cm^− 1^, indicating the presence of RB in the complex. This demonstrated that RB was interacting with the polymer [52]. These results suggest a clear interaction between RB and PVA in the RB@PVA matrix, as the FTIR spectrum of the physical mixture differs from the spectrum of the RB@PVA matrix. The interaction between PVA and RB led to a reduction in RB peak intensity. However, it is essential to note that there were no peaks in the FTIR spectrum of the RB@PVA matrix, suggesting the lack of chemical reactions between RB and PVA, leading to the creation of new covalent bonds. The interaction between PVA and RB is purely non-covalent. These results are consistent with the formation of hydrogen bonds between the OH groups in PVA and the C = O group in RB [38].

Mechanical testing of the RB@PVA matrix (Fig. 4C-E) was conducted using PVA or RB@PVA matrix rather than pure powders. The test aimed to confirm the interactions between PVA and RB. It was designed to provide more information on the RB@PVA matrix, though it does not represent the forces the materials will experience during application or manufacturing. Results (Fig. 4C-E) demonstrated that the material was stiffer when RB was added to the mixture, as the elastic modulus significantly increased compared to the pure PVA matrix. This result aligns with previous results indicating interactions between RB and PVA. Interestingly, the interaction between RB and PVA does not directly influence offset yield strength or elongation at break. These results are unsurprising, considering that hydrogen bonds are responsible for PVA mechanical properties [53]. The inclusion of RB will lead to the formation of new hydrogen bonds between the polymer and RB.

#### In vitro dissolution study of milled RB@PVA matrix

**Fig. 5:**
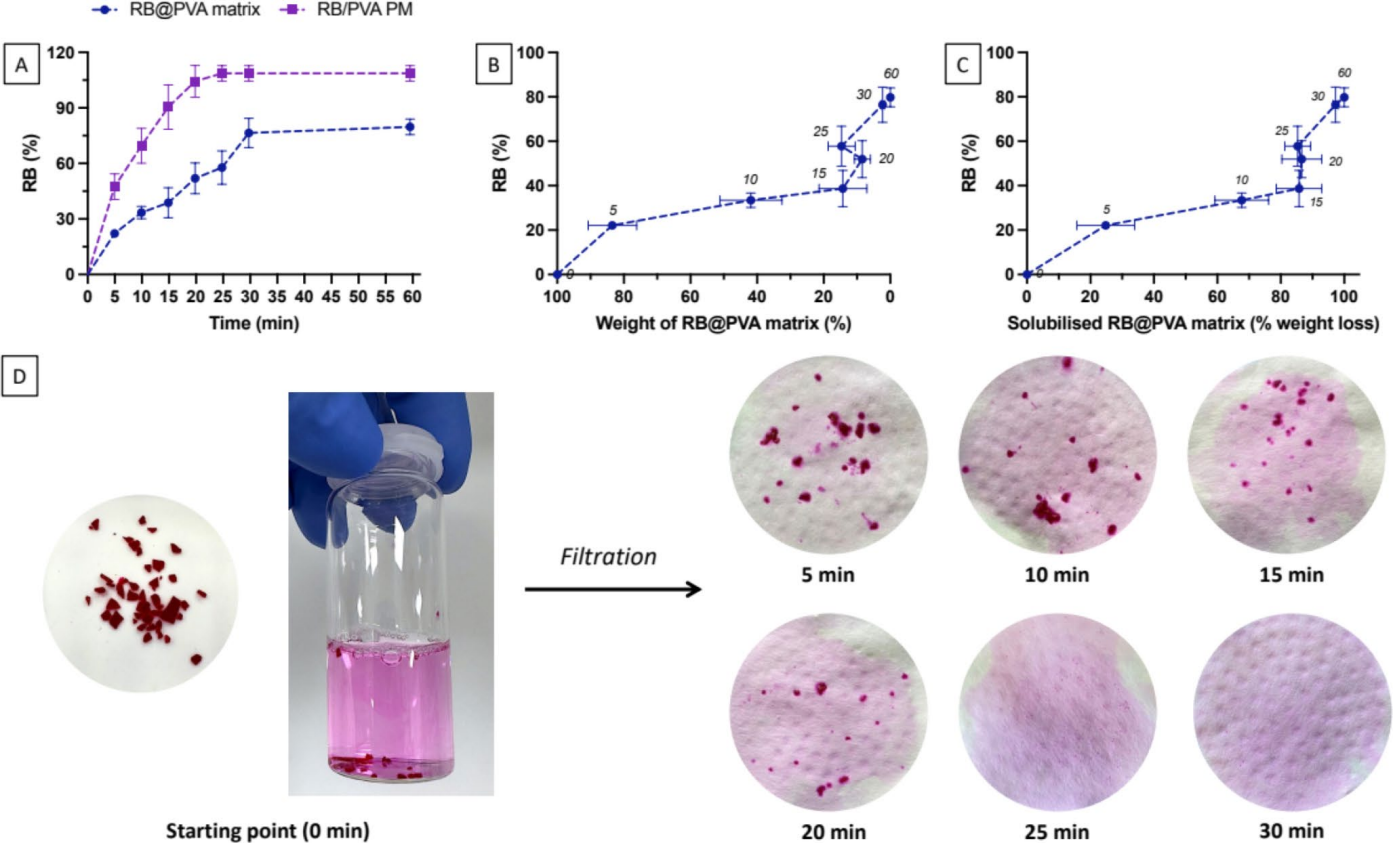
In vitro dissolution study of the milled Rose Bengal-loaded PVA (RB@PVA) matrix in buffer medium (pH 7.4, 37 °C). **(A)** Percentage RB released over time (min) from RB@PVA matrix compared to RB/PVA physical mixture. **(B)** Percentage of RB released relative to matrix weight loss. Corresponding time (min) is indicated above each symbol. **(C)** Correlation between RB release and matrix weight loss. Corresponding time (min) is indicated above each symbol. **(D)** Visual representation of residual RB@PVA particles on the filter at various time points (initial amount: 10 mg). Results are expressed as mean ± SD of four samples (*n* = 4)

Before incorporating the RB@PVA matrix into the implantable device, the release profiles of RB from the RB@PVA matrix and the RB/PVA physical mixture were evaluated over 60 min to understand their release kinetics. Results are displayed in Fig. 5.

The RB/PVA PM exhibited a rapid release (Fig. 5A). Approximately 70% (69.59 ± 9.43%) of RB was released within the first 10 min, reaching a plateau of around 90% (110.12 ± 14.33%) by 20 min. This fast-release behaviour is typical of physical mixtures, where RB is readily available on the surface or loosely associated with the PVA, allowing for immediate dissolution. Conversely, the RB@PVA matrix demonstrated a more controlled and sustained release. Initially, only 33.44 ± 3.29% of RB was released within the first 10 min, gradually increasing to 76.46 ± 7.96% by 30 min and around 79.78 ± 4.26% by 60 min. This slower release is proper for a matrix system where RB is embedded within the PVA matrix, leading to a more controlled diffusion and prolonged release profile.

An inverse relationship between the matrix’s weight and the amount of RB released was observed (Fig. 5B), parallel to the positive correlation between RB released and the amount of solubilised matrix (Fig. 5C). Interestingly, it can be seen that up until 25 min PVA and RB are released at similar rates. However, after that time, there are fluctuations in the sample weight due to potential PVA swelling before complete dissolution.

The observed variability in the release profile, indicated by the inter-replicate error, could be attributed to several factors. One significant factor is the particle size distribution of the milled RB@PVA matrix. Variability in particle size can lead to inconsistent surface area exposure to the buffer medium, resulting in fluctuations in dissolution and release rates. Larger particles may dissolve more slowly, while smaller particles dissolve more rapidly, contributing to the overall variability. Optimising the particle size distribution of the milled RB@PVA matrix is crucial to improve consistency in the release profile. This could be achieved through controlled milling processes and subsequent sieving to obtain a uniform particle size range. Narrowing particle size distribution will result in a more predictable and uniform dissolution rate [54]. Additionally, exploring different RB-to-PVA ratios is crucial for understanding their impact on the release profile. Although this study focused on a specific ratio, relevant insights can be gained from comparisons with existing research. For example, Hong et al. used a lower RB concentration of 0.28% w/w in a freeze-thawed PVA hydrogel. This study demonstrated that RB release reached equilibrium after approximately 48 h, likely due to the lower RB concentration, making the system suitable for short-term applications such as wound healing [55]. In contrast, our study, which utilised a higher RB concentration (5% w/w), demonstrated a prolonged release over 90 days. This comparison underscores the significant impact of RB concentration on release kinetics, with lower concentrations favouring quicker release and equilibrium and higher concentrations supporting sustained release over a more extended period.

Finally, Fig. 5D visually examines the progressive solubilisation of the RB@PVA matrix by presenting the amount remaining in the filter at each time point. Considering that no visible RB@PVA residuals were observed in the buffered medium at the end of the experiment, it is unclear why 100% RB release was not obtained. However, reflecting the demonstrated interaction between PVA and RB, it may be assumed that microscopic complexes of the two molecules, on the nanometer scale, formed upon matrix dissolution, impairing the full release of RB into the medium.

As demonstrated by the in vitro dissolution study, PVA plays a significant role in modulating the solubilisation behaviour of RB. While RB has a high solubility in water (100 g/L), the dissolution time increased to 30 min when incorporated into the PVA matrix. This delay is likely due to the interactions between RB and the PVA polymer network, which modulate the release and dissolution process. With its amphiphilic nature, PVA forms micelle-like structures and functions as a polymer surfactant, displaying a critical micellar concentration (CMC) in the range of 0.1-1 mg/mL, depending on hydrolysis [56]. The PVA concentration was 0.95 mg/mL during this experiment, within the CMC range. This micelle formation could encapsulate RB, contributing to the incomplete release observed in the medium. Moreover, PVA decreases the crystallinity of RB/PVA composite due to hydrogen bonding with RB, further stabilising the dye within the matrix [38, 57]. Literature suggests that RB may migrate to less polar environments, such as lipids, in aqueous solution, interacting with the hydrophobic groups of PVA. This interaction could further explain the incomplete release observed. These factors highlight the critical role of PVA in stabilising and modulating the release of RB [32, 44, 58].

### In vitro RB release study from RB@PVA matrix-loaded implants

As described previously, FDM was used to prepare reservoir-type implants containing a rate-controlling membrane composed of PCL and PLA [37]. Specifically, based on our earlier research, the 50PCL:50PLA combination was identified as particularly effective in achieving sustained and controlled drug release. Therefore, we focused exclusively on this combination in the current study. Previous studies have explored PCL combinations [59], but those membranes were not suitable for integration with 3D-printed PLA. This study included PLA in the membrane formulation to provide better thermal resistance for the 3D-printing process, as PCL has a low melting point (50–60 °C) [60].

**Fig. 6:**
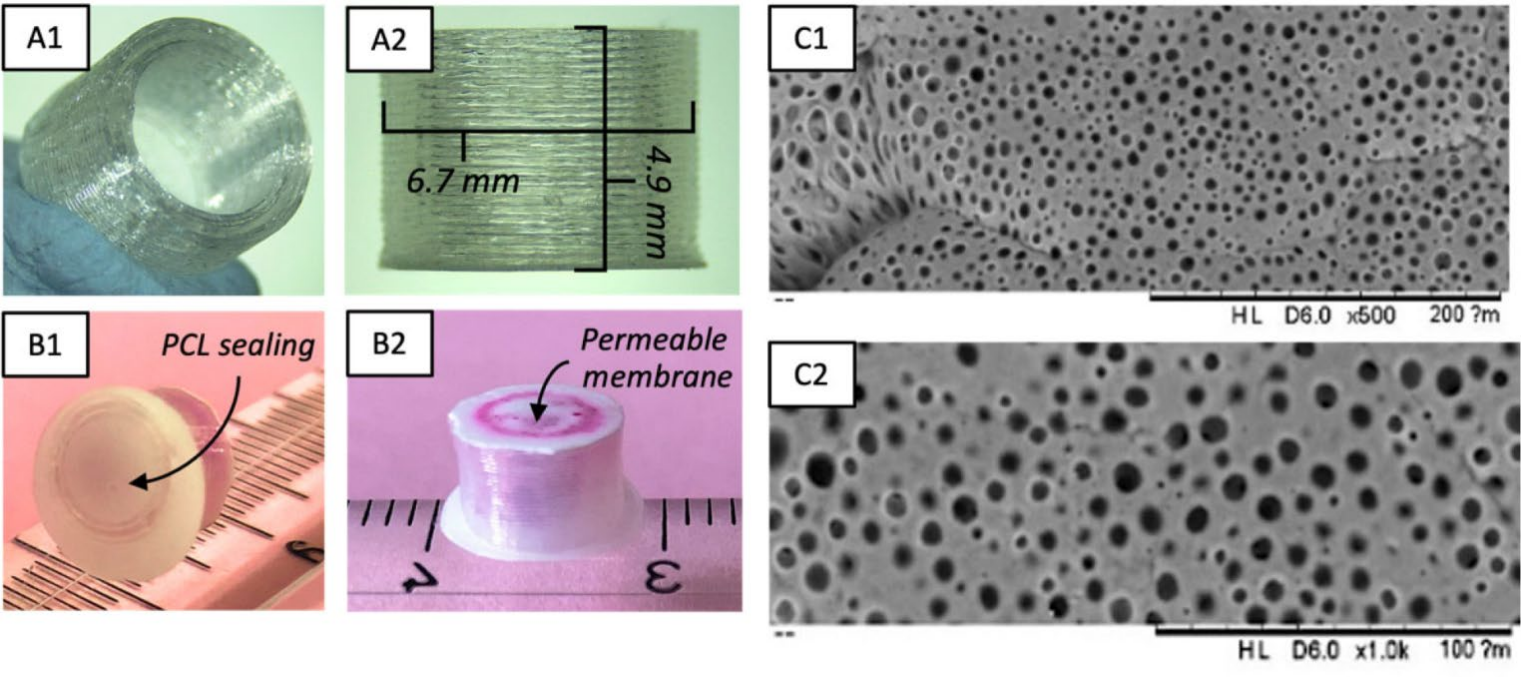
Rose Bengal-PVA (RB@PVA) matrix-loaded implant before in vitro release study. **A1-2.** Empty implant without PCL sealing. **B1-2.** Sealed RB@PVA matrix-loaded implant before the release study, showing PCL sealing **(B1)** and the permeable membrane **(B2)**. **C1-2** SEM micrographs of the permeable membrane before the release study (D1: scale bar: 0.2 mm, magnification: 500x; D2, D2: scale bar: 0.1 mm, magnification: 1.0kx)

**Fig. 7:**
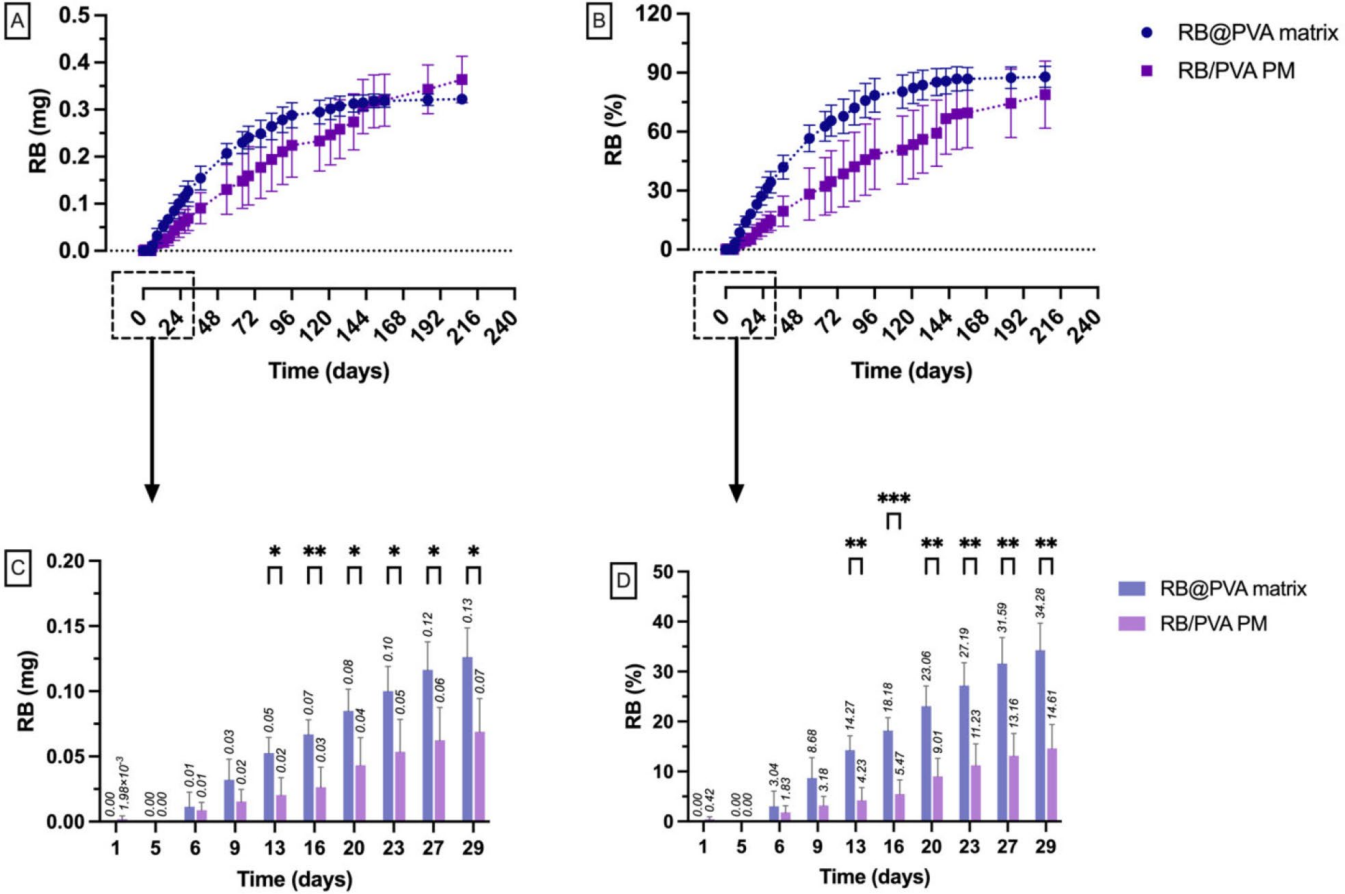
In vitro release profile of Rose Bengal (RB) from RB@PVA matrix-loaded implants compared to RB/PVA physical mixture (RB/PVA PM) implants in buffer solution (pH 7.4, 37 °C). **(A)** Amount (mg) of RB released over time. **(B)** Percentage of RB released over time. (**C-D**) Detailed view of RB release during the first 30 days. Results are expressed as mean ± SD of four samples (*n* = 4). Statistical analysis was performed using two-way ANOVA with the Geisser-Greenhouse correction and Fisher’s LSD test for multiple comparisons. Significance is indicated as *p < 0.05

Figure 6 shows the resulting implant and the attached rate-controlling membrane. Figures 6A-B and 7C depict the implant before starting the in vitro release experiment; particularly, Fig. 6A1-A2 illustrates the unloaded implant, evidencing the size and cylindrical cavity. On the other hand, Fig. 6B1-B2 illustrates the formulation-loaded implant, with the PCL cap seal in one extremity and the permeable membrane highlighted on the opposite extremity.

These devices were prepared with biocompatible and biodegradable polymers: PLA and PCL [61]. Therefore, these implants will not need to be removed after cargo release. Also, 3D printing allows clinicians to adapt the implant shape and drug cargo to the patient’s needs. This way, a customised implant could be prepared for a specific patient before surgery. In the past, several examples of matrix-type and reservoir-type implants [37, 62–66] have been prepared using 3D printing.

The RB@PVA matrix was incorporated into the implant and tested for in vitro release in a buffered medium (pH 7.4, 37 °C). As a control, the in vitro release was also performed for the implants loading RB/PVA as a physical mixture (RB/PVA PM) and free RB. Figure 7 illustrates the results of RB@PVA matrix and RB/PVA PM-loaded implants; the release profile of free RB-loaded implants is reported in the Supplementary Information (SI Fig. 1S).

The experiment lasted about 7 months and ended when a plateau phase was reached by the RB@PVA matrix-loaded implant (Fig. 7A-B). The influence of the implant in modulating the rate of RB release is readily evident, confirming the outcomes observed in the preceding publication [37]. Indeed, RB required only 30 min to achieve the maximum release when the RB@PVA matrix was placed in direct contact with the buffered medium; however, when loaded in the implant, the highest release amount was reached after 3 months (90 days). At this time, the cumulative amount of RB released was 0.28 ± 0.03 mg (75.84 ± 8.75%)— p-value ≤ 0.05 of time points precedent to 90 days vs. final time point and p-value > 0.05 of subsequent time points vs. final time point. Moreover, this result aligns with the amount of RB released when the matrix is directly suspended in the medium. Considering the rapid water-solubility of the RB@PVA matrix, the mechanism of drug release from the implant is consequent to water entrance through the permeable membrane, gradually solubilising the matrix, leading to drug release. Therefore, a diffusion mechanism governed by the porous membrane of the implant mainly controls the drug release rate without the influence of other processes (e.g., bulk erosion and degradation) [67, 68].

Notably, in the case of the RB@PVA-loaded implant, we did not observe a high degree of variability in the amount of RB released when the matrix was placed directly in contact with the buffer medium. Previously, variability was attributed to inconsistent surface area exposure due to particle size distribution, where larger particles dissolve more slowly and smaller particles more rapidly, contributing to fluctuations in the dissolution and release rates. This issue was circumvented by incorporating the matrix into the implant, leading to a more consistent release profile. This indicates that the implant’s design mitigates the need to optimise particle size distribution through controlled milling and sieving processes to achieve uniform dissolution rates. This observation is consistent with literature that discusses the role of controlled-release membranes in similar drug delivery systems. According to Siepmann and Peppas, the design parameters of the polymer structure, including its composition and geometry, significantly control the release kinetics, often independent of the drug particle size [69]. This principle is particularly relevant for systems like the one presented herein, where the membrane system design is dominant in ensuring controlled release. This supports our findings that the irregular particle size of the milled product does not adversely affect the release of RB from the implant, as the properties of the membrane primarily govern the controlled-release mechanism. Additionally, the review by Rambhia and Ma further supports this observation by demonstrating that polymer scaffolds and composite systems can be engineered to control drug release kinetics through formulation design, regardless of initial particle size [70]. Finally, this consistent release profile is a significant advantage and suggests that the implant system could benefit other particle-based drug delivery systems.

The exact mechanism is likely to control the release of RB from the physical mixture of RB/PVA. However, differences are evident compared to the RB@PVA matrix-loaded implant (Fig. 7). First, from Fig. 7A and B, it is clear that this formulation did not reach the plateau phase after 7 months, meaning that RB release was not complete. Statistical analysis showed that the highest amount of RB released was achieved after 5 months (day 149). At this time, 0.32 ± 0.06 mg of RB was released (69.00 ± 18.01%) (*p* ≤ 0.05) for time points before 149 days compared to the final time point, and a p-value > 0.05 for subsequent time points compared to the final time point. In the second instance, a slower drug release rate of RB/PVA PM compared to RB@PVA matrix was observed (Fig. 7), evident from day 13 (Fig. 7C-D), accompanied by increased experimental error. The slower drug release rates observed with the RB/PVA PM could be attributed to the heterogeneity of the formulation. Unlike the homogeneous structure of the RB@PVA matrix, the RB/PVA PM is composed of mixed RB and PVA powders, leading to an irregular distribution of RB powder within the PVA matrix. Consequently, unpredictable interactions may occur with the aqueous medium, where some RB powder is readily accessible and dissolves quickly. At the same time, others may be enclosed or foiled by PVA, delaying RB release. Moreover, PVA may be on its swells before dissolving in water. As mentioned earlier, RB can interact with the swollen PVA network, slowing the release process. Also, these swollen PVA gels could prevent free liquid permeation through the membrane, delaying the release process. These two processes can contribute to the variability in the obtained results. Furthermore, the physical mixture lacks the uniformity of the matrix, which was designed to facilitate controlled release through the matrix’s structure and composition. As a result, RB release from RB/PVA PM was characterised by irregular solubility and dispersion of the constituents, terminating in a slower, more heterogeneous release profile [71, 72].

**Fig. 8:**
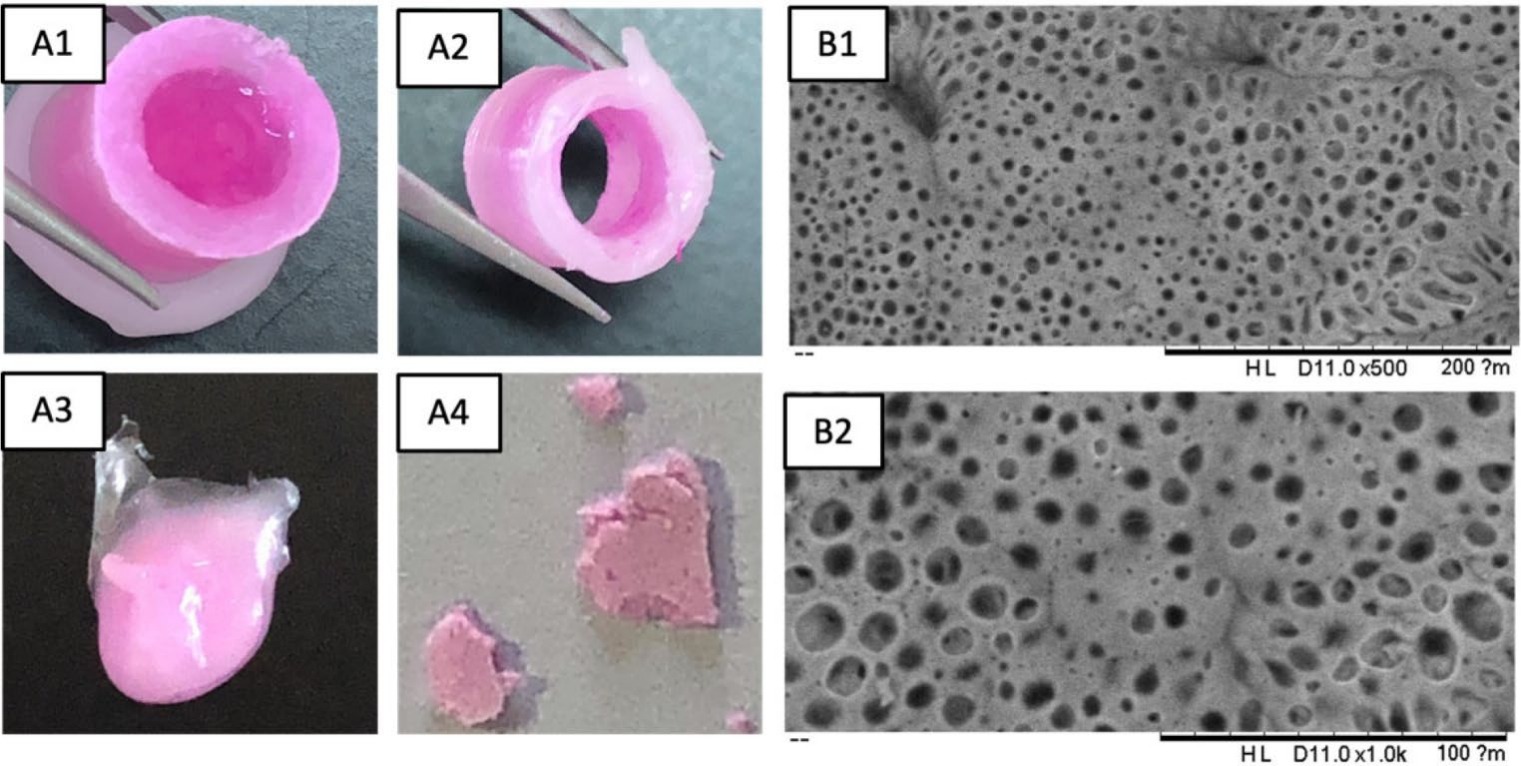
Rose Bengal-PVA (RB@PVA) matrix-loaded implant after a 7-month in vitro release study. **(A1)** residual RB@PVA matrix in the implant; **(A2)** cylinder structure after washing; **(A3)** removed PCL sealing; **(A4)** removed permeable membrane. (**B1-2**) SEM micrographs of the permeable membrane after the release study (B1: scale bar: 0.2 mm, magnification: 500x; B2: scale bar: 0.1 mm, magnification: 1.0kx)

All parts of the implant were disassembled at the end of the experiment and are shown in Fig. 8.

Figure 8A1 displays the implant before washing, where only the permeable membrane was detached. As discussed in this section, a jelly residue of RB@PVA matrix remained in the implant, even though it no longer contributed to drug release. This explains why a 100% RB release was not achieved during the study, thereby confirming the significant interaction between RB and PVA; these outcomes may also relate to the incomplete RB release obtained when the matrix is directly in contact with the medium. After washing, RB staining was still noticeable in the cylinder walls (Fig. 8A2) and is even visible in the PCL sealing (Fig. 8A3) and PCL/PLA permeable membrane (Fig. 8A4). This was expected considering the solubility properties of RB in solvents with a broad polarity and its logP [73]. In comparing the SEM images of the membrane before and after the release study (Figs. 6 and 8), changes in the pore structure and distribution were noted. Before the release study, the membrane’s pores were well-distributed, uniform in size, and had a consistent, rounded shape, which created a stable environment for controlled and steady RB release. After the release study, the pores appeared more irregular in size and shape, with some expansion and merging observed. While these structural changes suggest potential modifications to the membrane’s properties, the release study did not indicate significant fluctuations that would directly correlate with these changes. Therefore, while the changes in pore structure are notable, they do not seem to have considerably impacted the release profile under the conditions tested. These observations highlight the robustness of the membrane design in maintaining controlled RB release, even as the pore structure undergoes modifications during the release process.

### Cytotoxicity study on prostate cell lines

**Fig. 9:**
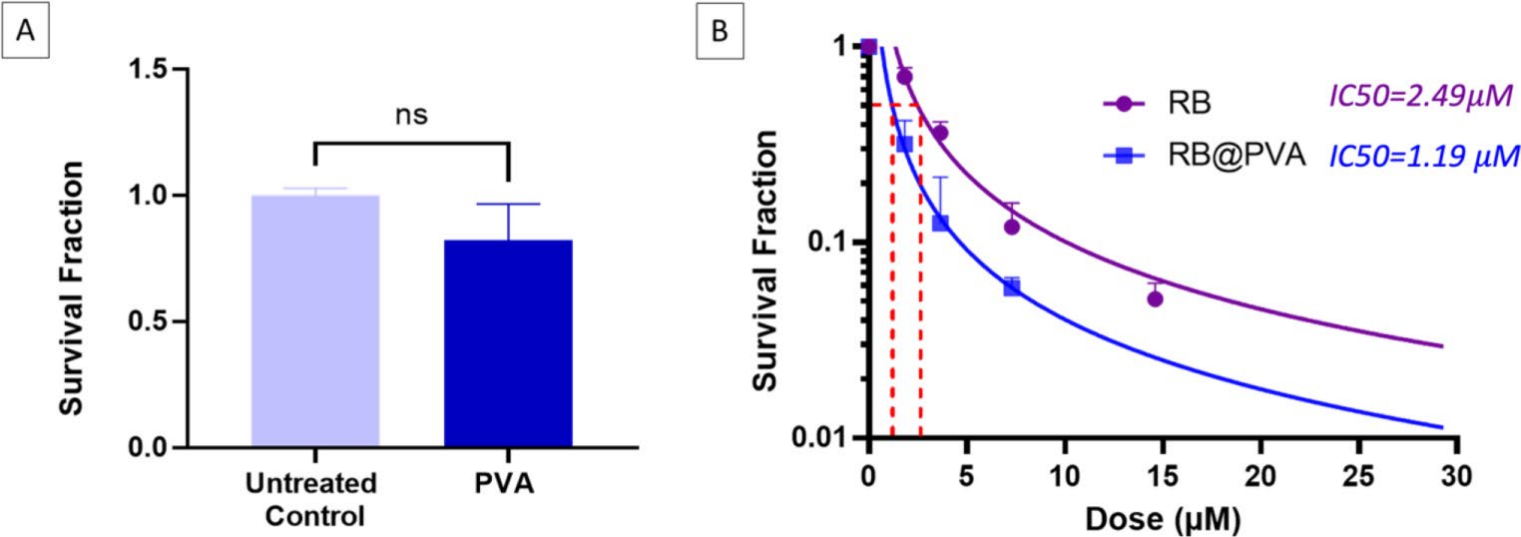
Cytotoxicity assay of free RB and RB@PVA matrix on PC-3 prostate cancer cells using clonogenic assays. **(A)** Survival efficiency of PC-3 cells treated with PVA at 30 µM. **(B)** Survival efficiency of PC-3 cells treated with different concentrations of free RB and RB@PVA matrix. Results are expressed as mean ± SD of three independent samples (*n* = 3). Statistical analysis was performed using a two-way ANOVA, with a highly significant (*p* < 0.0001) increase in direct cytotoxicity observed between RB@PVA matrix over free RB (*n* = 3)

In this investigation, we aim to evaluate the intrinsic cytotoxicity of the RB@PVA matrix on prostate cancer cells without light irradiation (Fig. 9).

As expected, PVA alone proved biocompatible on the PC-3 prostate cancer cell line (Fig. 9A); this outcome is consistent with existing literature that supports PVA as a biocompatible material, widely used in biomedical applications due to its non-toxic nature and excellent physical properties [25, 27]. A clonogenic assay was performed to assess the sustained anticancer effect, precisely 14 days post-treatment, of free RB and RB@PVA matrix. Both free RB and RB@PVA matrix produced a dose-dependent reduction in viability towards PC-3 cells, attributed entirely to the intrinsic cytotoxic properties of RB (Fig. 9B). The IC50 values for free RB and RB@PVA matrix were determined to be 2.49 and 1.19 µM respectively. The lower IC50 value of RB@PVA matrix suggests that the encapsulation of RB within the PVA matrix potentially increases the cytotoxic efficiency of RB, possibly due to improvements in cellular uptake [74, 75].

According to the release pattern of the RB@PVA matrix from the implant and the cell viability data, it is predicted that a therapeutic concentration will be achieved within a week and sustained for several months, crucial for sustained therapeutic applications, potentially reducing the frequency of treatments and improving patient compliance. However, it is essential to note that these results are derived from in vitro studies. In vivo studies are necessary to model and validate this effect in a more physiologically relevant context. Metabolism, immune response, and tissue interactions can significantly influence therapeutic outcomes. Moreover, the assay does not account for the additional cytotoxic potential from the photodynamic effect of the RB@PVA matrix. When exposed to light, RB can generate reactive oxygen species (ROS), which can cause further damage to cancer cells. Thus, the combined intrinsic and photodynamic cytotoxicity of the RB@PVA matrix could potentially lead to a more pronounced therapeutic effect, making it a promising candidate for combined cancer therapy.

## Conclusions

This study reported developing and characterising a novel drug delivery system comprising RB@PVA matrix within 3D printed PCL/PLA implants for local and sustained cancer treatment. The encapsulation of RB within the PVA matrix achieved a loading efficiency of 77.34 ± 1.53%. Including the implant facilitated the controlled drug release over 90 days, maintaining effective drug concentrations at the tumour site. The RB@PVA matrix demonstrated cytotoxicity against PC-3 prostate cancer cells, with an IC50 value of 1.19 µM compared to 2.49 µM for free RB, indicating enhanced efficacy. Physicochemical and mechanical characterisations revealed interactions between RB and PVA, and the mechanical testing showed increased stiffness of the PVA matrix upon RB incorporation. The in vitro dissolution and release studies confirmed the ability of the implant to provide sustained RB release, reducing the need for frequent drug administration and potentially improving patient compliance. The innovative RB@PVA matrix-loaded implants significantly advance local cancer therapy by providing sustained drug release, enhancing therapeutic efficacy, and minimising systemic toxicity. These findings suggest that implantable devices could reduce the frequency of painful administrations associated with cancer treatments and improve patient outcomes. However, to fully validate the in vitro results and explore the clinical potential of this novel drug delivery system, future in vivo studies are warranted. These studies are essential for assessing implant materials’ long-term biocompatibility, degradation behaviour, and overall safety within a physiological environment. In vivo research will also be crucial for determining any potential adverse reactions, the material degradation rate, and the implants’ sustained efficacy over extended periods. In summary, this research presents a promising approach for enhancing cancer treatment through localised and sustained drug delivery, potentially leading to more effective and less toxic therapeutic options for patients. Integrating in vivo studies in future research will be critical to advancing this system towards clinical application.

## Electronic supplementary material

Below is the link to the electronic supplementary material.

Supplementary Material 1

## Data Availability

The datasets generated during and analysed during the current study are available from the corresponding authors upon reasonable request.
