# Supplementary material for: Evaluating the efficacy of Rose Bengal-PVA combinations within PCL/PLA implants for sustained cancer treatment
Source: Drug Deliv Transl Res. 2024 Sep 23;15(5):1770–85. doi: 10.1007/s13346-024-01711-w (PMC11968511; doi:10.1007/s13346-024-01711-w)
Supplement: Supplementary file 1 — Supplementary Material 1 [file 13346_2024_1711_MOESM1_ESM.pdf]

Supplementary Information

Evaluating the Efficacy of Rose Bengal-PVA combinations within PCL/PLA Implants for Sustained Cancer Treatment

Sara Demartis[a], Camila J. Picco[b], Eneko Larrañeta[b*], Anna Korelidou[b], Rayhanul Islam[b], Jonathan A. Coulter[b], Paolo Giunchedi[c], Ryan F. Donnelly[b], Giovanna Rassu[c], Elisabetta Gavini[c*].

[a] Department of Chemical, Physical, Mathematical and Natural Sciences, University of Sassari, Sassari, 07100, Italy

[b] School of Pharmacy, Queen's University Belfast, Belfast BT9 7BL, United Kingdom

[c] Department of Medicine, Surgery and Pharmacy, University of Sassari, Sassari, 07100, Italy

* Corresponding authors: Eneko Larrañeta (e.larraneta@qub.ac.uk), Elisabetta Gavini (eligav@uniss.it)

In vitro dissolution study of milled RB@PVA matrix

For the evaluation of the dissolution profile of milled RB@PVA matrix, 10 mg were dispersed in 10 mL of buffer medium (pH 7.4) and incubated at 37°C in an orbital incubator (SKI 4 Shaker Incubator, Argo Lab, Carpi, Italy) at 40 rpm. At each predetermined time point (5, 10, 15, 20, 30, 60 min), the entire medium was collected and filtered under vacuum using a Büchner funnel with a cellulose membrane filter with pore size in the magnitude of micrometres. The isolated residual RB@PVA matrix was frozen at -80°C, then freeze-dried (LIO-5P 4K, Milan, Italy) for 7 h. Afterwards, the residual RB@PVA matrix was weighed. The percentage of matrix weight loss and the correspondent solubilised matrix were calculated at each time point: details on calculations are reported below:

Time (min)	Sample	Matrix weight (mg)	Filter weight (g)	Total weight (g)	Matrix on the filter (mg)	Solubilised matrix (mg)
5	A	10.2	0.2112	0.2179	6.7	3.5
	B	10.5	0.2204	0.2292	8.8	1.7
	C	10.6	0.1761	0.1857	9.6	1
	D	10	0.1793	0.1869	7.6	2.4
10	A	10.4	0.2082	0.2117	3.5	6.9
	B	10.2	0.166	0.1713	5.3	4.9
	C	9.9	0.1771	0.1794	2.3	7.6
	D	10.5	0.1843	0.1885	4.2	6.3
15	A	9.6	0.2066	0.2072	0.6	9
	B	10.3	0.2145	0.2192	4.7	5.6
	C	11	0.1852	0.1874	2.2	8.8
	D	9.7	0.1861	0.1877	1.6	8.1
20	A	10.9	0.2141	0.2151	1	9.9
	B	10.6	0.1806	0.1817	1.1	9.5
	C	10.4	0.175	0.1756	0.6	9.8
	D	10.2	0.1887	0.1908	2.1	8.1
25	A	10.3	0.1889	0.1908	1.9	8.4
	B	10.6	0.1795	0.1806	1.1	9.5
	C	10.2	0.1804	0.1806	0.2	10
	D	9.9	0.186	0.1875	1.5	8.4
30	A	9.7	0.1734	0.1736	0.2	9.5
	B	10.4	0.1773	0.1777	0.4	10
	C	10.3	0.1829	0.1832	0.3	10
	D	10	0.1816	0.1818	0.2	9.8

In vitro RB release study from free RB-loaded implants: results

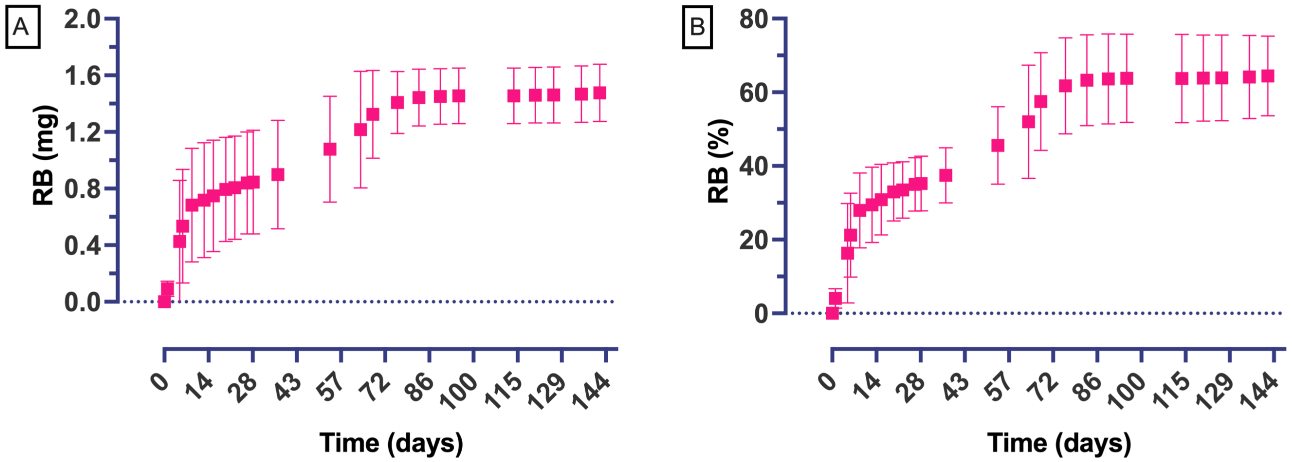

Fig. S1 *In vitro* release profile of free RB implants in buffer solution (pH 7.4, 37°C). **A.** Amount (mg) of RB released over time. **B.** Percentage of RB released over time
